# Potential Pasture Nitrogen Concentrations and Uptake from Autumn or Spring Applied Cow Urine and DCD under Field Conditions

**DOI:** 10.3390/plants5020026

**Published:** 2016-06-13

**Authors:** Jim Moir, Keith Cameron, Hong Di

**Affiliations:** Soil Science Department, Lincoln University, Christchurch, Lincoln 7647, New Zealand; keith.cameron@lincoln.ac.nz (K.C.); hong.di@lincoln.ac.nz (H.D.)

**Keywords:** plant nitrogen, soil nitrogen, nitrification inhibitor, dicyandiamide (DCD), grazed pasture, urine, dairy

## Abstract

Nitrogen (N) cycling and losses in grazed grassland are strongly driven by urine N deposition by grazing ruminants. The objective of this study was to quantify pasture N concentrations, yield and N uptake following autumn and spring deposition of cow urine and the effects of fine particle suspension (FPS) dicyandiamide (DCD). A field plot study was conducted on the Lincoln University dairy farm, Canterbury, New Zealand from May 2003 to May 2005. FPS DCD was applied to grazed pasture plots at 10 kg·ha^−1^ in autumn and spring in addition to applied cow urine at a N loading rate of 1000 kg·N·ha^−1^, with non-urine control plots. Pasture N ranged between 1.9 and 4.8% with higher concentrations from urine. Results indicated that urine consistently increased N concentrations for around 220 days post deposition (mid December/early summer) at which point concentrations dropped to background levels. In urine patches, pasture yield and annual N uptake were dramatically increased on average by 51% for autumn and 28% for spring applied urine, in both years, when DCD was applied. This field experiment provides strong evidence that annual pasture N uptake is more strongly influenced by high urine N deposition than pasture N concentrations. FPS DCD has the potential to result in very high N uptake in urine patches, even when they are autumn deposited.

## 1. Introduction

Nitrogen (N) is essential for pasture growth, and is often the key limiting nutrient in grazed grasslands [1]. The urine patch of the grazing ruminant plays a critical role in N cycling and losses from pasture systems due to high N concentrations in the urine and labile carbon (C) additions [2]. As pasture growth in the animal urine patch typically responds to the high N loading, grazed pastures typically form a visual mosaic of “lumpy” urine patches and flat “inter-urine” areas. In an intensively grazed temperate dairy pasture, urine depositions would typically cover ≈25% of the paddock or farm area on an annual basis [3].

The urine patch of the grazing animal has been identified as the main source of N leaching losses in grazed grasslands [4,5]. The N loading rate of cow urine patches is often between 700 and 1200 kg·N·ha^−1^, which is often stated as being in excess of potential pasture N uptake during the growing season [6]. As such, the potential of N leaching losses from cow urine patches is high, particularly during the winter period when soils are draining [7]. Considerable recent research has focused on reducing N leaching losses and gaseous N emissions from the urine patch of the grazing dairy cow [8]. A key development in this field has involved the application of the nitrification inhibitor dicyandiamide (DCD) to grazed pastures to reduce the production and leaching of nitrate from the soil [8,9]. DCD inhibits the first stage of nitrification, the oxidation of NH_4_^+^ to NO_2_^−^, by rendering the bacteria’s enzymes ineffective [8]. The application of DCD as a fine particle suspension (FPS) at 10 kg·ha^−1^ in autumn (May) and late winter (August) was shown to decrease NO_3_-N leaching from 134 kg·N·ha^−1^·year^−1^ to 43 kg·N·ha^−1^·year^−1^ (equivalent to a 68% reduction) from the dairy cow urine N applied in the autumn (May) at the rate of 1000 kg·N·ha^−1^ [10]. A field study has also demonstrated pasture yield and N uptake increases, by 29 and 29%, respectively, by applying DCD in this manner [11].

However, the dynamics of pasture N concentrations in grazed pasture systems are not well understood, particularly in terms of temporal changes in urine patch pasture N and potential N uptake. Closely associated with these questions is what effect DCD may have on pasture −N concentrations and annual N uptake. Although some data indicating increased N uptake with DCD application, has been presented by Moir *et al.* [11], substantial further data in this field is required to better understand N cycling in temperate grazed pasture systems. The objective of this study was to determine the effects of cow urine and DCD on pasture N concentrations, uptake and yield for autumn and spring deposited urine under field conditions.

## 2. Results

### 2.1. Pasture N Concentrations

**Figure 1:**
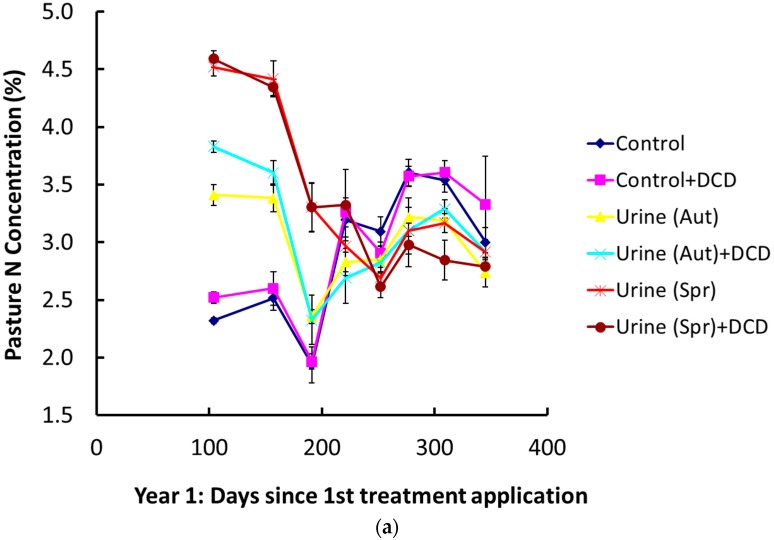

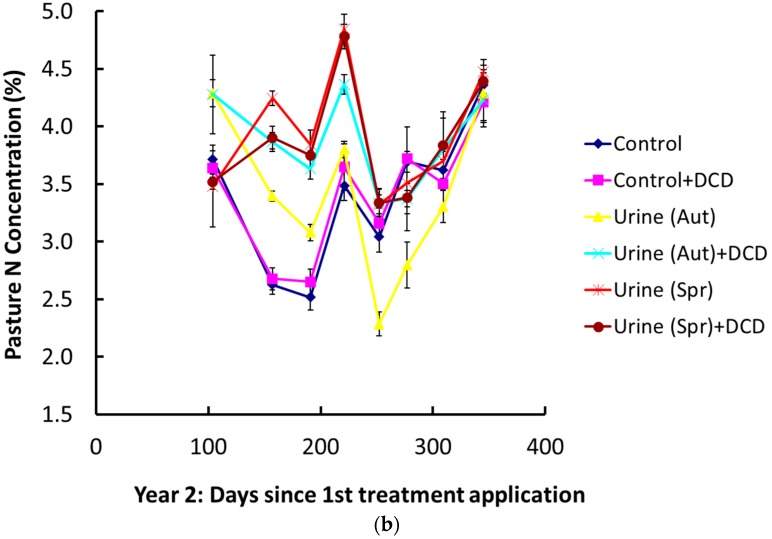
Mean pasture N concentrations for all treatments in: (**a**) Year 1 (2003/04 season); (**b**) year 2 (2004/05 Season). Experiment start date (Day 0) is 7 May in both years. Error bars represent ± 1 SEM, *n* = 4.

Non-urine treatments had lower mean annual pasture N concentrations than in all urine treatments (*p* < 0.001; Table 1). Mean annual pasture N concentrations in urine patches increased in autumn in both years as a result of DCD application (*p* < 0.001). The largest difference between autumn urine patches was in year 2, where DCD treated patches had mean annual N concentrations 0.45% higher than non-DCD treated patches. Without DCD, spring urine patches had higher N concentrations than autumn patches (*p* < 0.001). The same trend was observed where DCD was applied (*p* < 0.05), but N concentration differences were small. On a harvest basis, large differences in pasture N concentrations were observed between treatments for the first 200 days of the experiment in year 1 (Figure 1a). For the first 170 days, the highest N concentrations were in the order of spring urine patches (4.1% N) > autumn urine patches (3.3% N) > no urine (2.4% N). Only small treatment differences were apparent for the remainder of the growing season, with N concentrations varying between 2.7% and 3.6% N.

In year 2, differences in pasture concentrations due to treatment were less clear. Pasture in autumn applied urine patches (no DCD) generally had low N concentrations beyond 180 days post urine application, and had the lowest N concentrations of all treatments between days 220–320 (Figure 1b). N concentrations were high for spring urine patches and were low for non-DCD and DCD treatments, and also the autumn urine DCD treatment. The range of pasture N concentrations was wider in year 2, ranging from 2.3% to 4.8%. All treatments increased in N concentration, from around 3.2% to 4.2%, between days 250 and 350.

### 2.2. Yield

Total annual DM production across all treatments ranged from 11,644 kg·DM·ha*^−^*^1^ (control, year 1) to 28,089 kg·DM·ha*^−^*^1^ (Autumn urine + DCD, year 2) (Table 1). All yields were significantly higher in year 2 than year 1 (year 1: 14,654, year 2: 19,394 kg·DM·ha*^−^*^1^; *p* < 0.001). DCD treated urine patches had much higher yields than non-treated, in both autumn and spring patches, and in both years (*p* < 0.001, Table 1). Those yield increases were consistent at most harvests, throughout the 12 month growing season. For non-urine treatments, DCD treated pasture yielded 6% higher in year 2.

Within years, autumn urine patches yielded 47% higher (*p* < 0.001, Table 1) in year 1 where DCD was applied. A similar trend was observed for spring urine patches, with a 24% yield increase (*p* < 0.001) with DCD application. In year 2, DCD treated autumn urine patches yielded 37% higher, and 32% higher in spring urine patches with DCD. Non-DCD autumn urine patches out yielded spring patches in year 1, but not in year 2 (*p* < 0.01).

### 2.3. N Uptake

Large differences in annual pasture N uptake were observed between treatments and between years (Table 1). Values ranged from 342 (control, year 1) to 1080 kg·N·ha*^−^*^1^ (Autumn Urine+DCD, year 2). Mean N uptake was greater (*p* < 0.001) in year 2 (681 kg·N·ha*^−^*^1^) than in year 1 (462 kg·N·ha*^−^*^1^).

In year 1, autumn urine patches took up 52% more N where DCD was applied (*p* < 0.001), and spring patches 27% more N (*p* < 0.001). Year 2 was very similar, with autumn urine patches taking up 53% more N where DCD was applied (*p* < 0.001), and spring patches 27% more N (*p* < 0.001). For non-urine treatments, DCD treated pasture took up 7% more N (*p* < 0.05) in year 2. Spring urine patches had significantly higher annual N uptake than autumn patches where no DCD was applied (*p* < 0.001).

### 2.4. Climate

**Figure 2:**
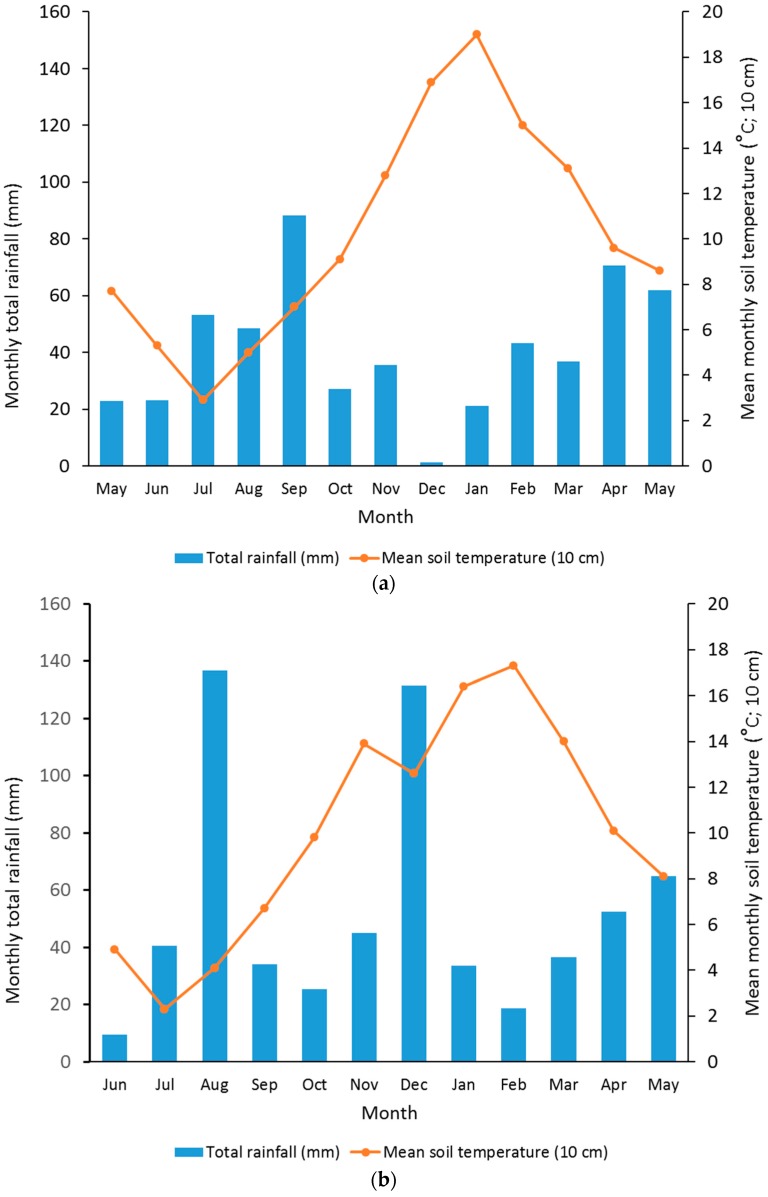
Mean monthly rainfall and 10 cm soil temperature in: (**a**) Year 1 (2003/04 season); (**b**) year 2 (2004/05 Season).

Annual rainfall was higher in year 2 (629 mm) than in year 1 (511 mm) (Figure 2). December of year one had very low rainfall (Figure 2a), while August and December of year 2 had very high rainfall (Figure 2b). Soil temperatures were very similar in both years (Figure 2), except in December of year 2, where temperatures dropped significantly (Figure 2b).

## 3. Discussion

### 3.1. N Concentrations

The objective of this study was to quantify pasture N concentrations and uptake in autumn and spring deposited urine patches and also the effects of DCD. Results from this study clearly show that urine patches caused increases in pasture N concentrations in the order of 2% above controls for more than 100 days. N concentrations in urine-affected pasture did not reduce to background concentrations (control) until around 220 days (mid December/early summer) after the autumn urine applications. Interestingly, this coincided with the N concentrations of spring deposited urine patches dropping to background concentrations in mid-December. The application of DCD had mostly minor effects on pasture N concentrations.

Interestingly, the pasture N concentrations in urine patch treatments were often very similar, regardless of whether the patch received DCD or not (see Section 4.1). This result suggests that for cow urine sourced N, peak pasture N concentrations were restricted to a maximum of around 4.8% N at this site, regardless of the likelihood of there being very high soil mineral N concentrations in the plant root zone. Given the very high N loading in the urine patch, it is therefore extremely unlikely that pastures of this type would ever exceed 4.8% N at the time of grazing.

These results strongly indicate that elevated N concentrations in cow urine patch pasture do not persist for the entire growing season, regardless of whether the urine was deposited in the autumn or spring. Instead, the duration of elevated pasture N concentrations seems to be driven by the onset of seasonal (spring and early summer) conditions and the associated period of high pasture N demand and growth rates, at least for autumn and spring deposited urine. This is a curious result, which suggests that luxury N uptake may not be occurring for half of the growing season, even when a high loading rate of 1000 kg·N·ha^−1^ has been applied.

Comparative literature on temporal fluctuations of pasture N concentrations in urine patches is scarce. However, the values reported here are in close agreement with those reported [11,12].

### 3.2. Yield

Pasture yield was strongly influenced by urine deposition, and DCD application, in both years. Large increases in yield resulted from urine deposition, and these increased further after applying DCD. Urine application increased annual yield by around 4 T DM·ha^−1^ (30%) above the non-urine treatments, while DCD treated patches yielded 10 T DM·ha^−1^ (76%) more. DCD treated urine patches consistently yielded significantly more DM on an annual basis compared to non-treated patches, in both seasons, and in both years. This strongly demonstrates the effect of DCD on soil urine N retention, and the subsequent increase in plant-available N for pasture growth.

Di and Cameron [8,10,13] reported similar pasture DM yield increases in urine patches following DCD application, in the region of 30%. The level of DM response also varied between years, which likely resulted from climatic variability. However, annual DM production mostly remained in the region of 20% above controls for both of the DCD treatments in those studies. In a four-year grazed pasture experiment, Moir *et al.* [11] reported a mean increase of 29% in urine patches where DCD was applied. Carey *et al.* [14] have also reported field pasture responses to DCD application.

### 3.3. N Uptake

Annual pasture N uptake differed dramatically between treatments in this experiment. Although N uptakes were lower in year 1 than year 2, a strong and consistent trend of much higher N uptake in DCD-treated than non-treated patches was observed. In year 2, DCD treated urine patch pasture took up 1030 kg·N·ha^−1^, which marginally exceeds the large N loading of 1000 kg·N·ha^−1^ applied as the treatment. This is a surprising and important result, which indicates that regardless of whether a cow urine patch is deposited in the autumn or winter, the pasture area affected by urine has the potential to take up all of the 1000 k·N·ha^−1^ in the urine, if the patch is treated with DCD. This has not previously been reported in the literature. It should also be noted that this result occurred in a year with excessively high rainfall events in the months of August and December 2004 (Figure 2b). In contrast, non-DCD treated patches only took up 61% of applied N on average, and in year 1 only took up 39% of autumn applied urine.

In itself, this result has major implications for reducing N loss for intensively grazed temperate pastures. As urine is the key source of N loss for such systems, DCD application has been shown to substantially increase N uptake in the urine patch, in one year/season to the point where all urine N has been assimilated by the pasture.

The urine patch of the dairy cow has been identified as the main source of N leaching loss from grazed pasture systems [7]. Similar trends in urine N uptake have been reported [11] on poorly drained heavy clay soils. These data also support the findings of Monaghan *et al.* [15], and Di and Cameron, who have reported the effects of DCD on the rate of nitrification in soil [16] and the subsequent reduction of N leaching loss [8,9,10,13].

Further research is required to reinforce the findings of these experiments. Such research should be conducted under grazed pasture conditions, and include cow urine deposited in several different months of the year, to observe in more detail the temporal effects of urine deposition and DCD under field conditions. Additionally, 15N studies could provide more detail on plant N uptake and soil N forms.

## 4. Experimental Section

### 4.1. Site Description and Farming System

The trial was conducted on the Lincoln University dairy farm, 15 km SW of Christchurch, New Zealand from May 2003 to May 2005. This farm was converted from a sheep farm to dairying in 2001. The mean annual maximum and minimum temperatures measured on the farm are, respectively, 17 and 4 °C, with an average annual rainfall of 666 mm, which is supplemented with approximately 500 mm of irrigation per annum. Stocking rate is 4.3 cows·ha^−1^ (2005), producing 427 kg·MS·cow^−1^ or 1720 kg·MS·ha^−1^ (2004/05 season). Stocks are grazed outdoors at all times, with a main diet of grazed pasture supplemented with pasture silage when necessary to maintain cow energy intake. Pastures were perennial ryegrass/white clover based.

The soil type at the trial site is a free-draining Paparua sandy loam (Immature Pallic soil [17]); Udic Haplustepts [18], with a 25-cm fine sandy loam Ah horizon. Soil chemical properties at the trial site are presented in Table 2. The current fertiliser applied to the farm includes maintenance dressings of 530 kg·ha^−1^·year^−1^ of single superphosphate (0:9:0:12) (N:P:K:S) as split spring/autumn dressings, plus 8 strategic dressings of urea at 25 kg·N·ha^−1^ (54 kg·urea·ha^−1^) throughout the season. This represents a total nutrient input of 200:48:0:60 kg·ha^−1^·year^−1^ from fertilizer.

A bulked soil sample (60 cores, 0–75 mm × 25 mm diameter) was taken from the general trial area in April 2003 and analyzed for soil fertility status. Results are presented in Table 2. Soil pH was measured at a water:soil ratio of 2.5:1 [19]. The method of Olsen *et al.* [20] was used to measure plant available soil P. Extractable soil sulphate was determined by the method of Searle [21]. Soil extractable cations were measured by method of Schollenberger and Simon [22]. A modified method of Waring and Bremner [23] and Keeney and Bremner [24] was used to measure soil mineralizable N.

### 4.2. Trial Design and Treatment Application

The trial area consisted of sixteen 4 m^2^ plots in a randomized design. All plots were situated in a typical paddock and irrigated as part of the normal management practices of the Lincoln University dairy farm. Grazing stocks were excluded. Plots were fenced off for more than eight months before trial commencement to remove any carry over effects of animal urine deposition. Within a plot a 0.5 m^2^ area was separated from the rest of the plot using a round sheet steel frame, which was inserted vertically into the soil to a depth of 20 cm. This area was used for the urine patch treatment within each plot, and the steel frame prevented lateral movement of urine after application. After year one, the steel frames were installed into a fresh area of the plot so that the same area did not receive urine twice, which is an unlikely event that would create an artificial N loading regime. Old urine areas were abandoned.

Treatments were first applied to the plots in early May 2003. Treatments were control; control + DCD; autumn urine; autumn urine + DCD; spring urine, spring urine + DCD, replicated four times. Simulated dairy cow urine patches were applied at the field site immediately prior to the DCD treatment application. Cow urine was collected from the dairy herd while cows were being milked, and was bulked. At the field site the cow urine (N content = 10 g·N·L^−1^) was then applied to the plots by pouring into the steel frame area, so that 5.0 L of cow urine was uniformly applied over an area of 0.50 m^2^ for a period of 10 s. This resulted in a total N loading rate of 1000 kg·N·ha^−1^ in the urine patches, which is the accepted mean value of N loading in the dairy cow urine patch [2,4,7,25]. Pasture N concentrations, yield and pasture shoot N uptake were then measured over the 12 month season. The simulated urine patch treatments were applied to respective treatments in May 2003, August 2003, May 2004 and August 2004. Urine was applied to new pasture areas at each treatment to prevent any areas receiving “double” applications.

Dicyandiamide (DCD) was applied to the DCD treatment plots as a fine particle (<70 µm) suspension (FPS) (DCD:water ratio = 100 g:1.5 L) at a rate of 10 kg·DCD·ha^−1^, in autumn (early May) and spring (early August) in both years. This fine particle suspension methodology of DCD application to pasture was developed to maximize spatial coverage of the compound, thereby increasing the compounds effectiveness on treating nitrifying bacteria in the soil [10]. Pastures were short (<1500 kg·DM·ha^−1^) and were freshly mowed at the time of application (<5 days post mowing). The “control” plots did not receive DCD at any time. Immediately following the DCD application 10 mm of irrigation was applied to wash DCD particles off pasture and into the soil matrix. For further detail of the development of the DCD nitrification inhibitor technology for application in grazed pasture systems readers are directed to [10]. Non-urine treatments (control and control + DCD) also received urea fertilizer at all 8 harvests, at a rate of 20 kg·N·ha^−1^.

### 4.3. Pasture Harvests

Pasture dry matter (DM) production was measured by method of dry matter cuts using quadrats of known area. The cuts were taken near the day that the dairy herd grazed the surrounding paddock, and for every grazing rotation of the farm over the 12-month season. At each harvest all treatments were harvested to a standard residual of 1500 kg·DM·ha^−1^ using electric clippers and a 0.25 m^2^ quadrate. This is the standard pasture residual level obtained on the Lincoln University dairy farm throughout the season. Quadrates were randomly placed on the non-urine treatments. Other areas of the plot were also harvested at the same time and the pasture discarded. All pasture samples were weighed fresh, mixed and visually assessed for clover content, then dried at 70 °C for 48 h and reweighed to calculate dry matter yield.

### 4.4. Herbage Analysis

All pasture samples were analyzed for total N content. Finely ground (1 mm) oven-dried (70 °C) herbage and total N was determined using Near Infra-Red Spectroscopy (NIRS) (NIRSystems 5000 monochromator, Foss-NIRSystems, Silver Spring, MD, USA) using equations generated with WinISI software (v1.50, Infrasoft International, ISI, Port Matilda, MD, USA).

### 4.5. Data Analysis

All data was compared on an annual basis for autumn and spring urine patches both with or without treatment with DCD, and controls. Data was analyzed on an individual sample basis. Annual pasture N uptake was calculated by multiplying yield by concentration on an individual sample basis. All data sets were statistically analyzed to test for treatment, year and harvest effects by conducting an analysis of variance (ANOVA) using GenStat 16.1 (Lawes Agricultural Trust, Rothamsted, UK). Normality and homogeneity of variance in the data was assessed during this analysis. Treatments were compared within non-urine, autumn urine and spring urine treatments. Autumn and spring urine treatments were also compared, using orthogonal contrasts.

## Figures and Tables

**Table 1:** Annual mean values of pasture nitrogen (N) concentrations, dry matter (DM) yields and pasture shoot N uptakes.

Treatment	Herbage N (%)		Yield (kg·DM·ha^−1^)		N Uptake (kg·N·ha^−1^)	
	Year 1	Year 2	Year 1	Year 2	Year 1	Year 2
Control	2.93	3.38	11,644	14,633	342	459
Control + DCD ^1^	3.00	3.40	11,850	15,548	352	493
*Treatment*	ns		ns	*	ns	*
*Year*	***		***		***	
*TreatmentxYear*	ns		*		*	
*LSD (5%)* ^2^	0.142		868.8		32.8	
Urine (Aut)	2.99	3.41	12,721	20,465	393	707
Urine (Aut) + DCD ^1^	3.04	3.86	18,738	28,089	599	1080
*Treatment*	***		***		***	
*Year*	***		***		***	
*TreatmentxYear*	***		ns		*	
*LSD (5%)* ^2^	0.193		2014.4		79.8	
Urine (Spr)	3.37	3.93	17,287	19,946	582	776
Urine (Spr) + DCD ^1^	3.32	3.86	21,499	26,292	740	986
*Treatment*	ns		***		***	
*Year*	***		**		***	
*TreatmentxYear*	ns		ns		ns	
*LSD (5%)* ^2^	0.158		2280.0		86.6	
Urine (Aut) *vs.* Urine (Spr) Contrast:						
*Treatment*	***		**		***	
*Year*	***		***		***	
*TreatmentxYear*	ns		**		ns	
*LSD (5%)* ^2^	0.104		1693.6		61.6	
Urine (Aut)+DCD *vs.* Urine (Spr)+DCD Contrast:						
*Treatment*	*		ns		ns	
*Year*	***		***		***	
*TreatmentxYear*	*		ns		*	
*LSD (5%)* ^2^	0.114		2595.2		103.2	

^1^ DCD rate = 10 kg·ha^−1^, applied in both Autumn (May) and Spring (August). ^2^ LSD between treatments. ns not significant, * significant at *p* < 0.05, ** significant at *p* < 0.01, *** significant at *p* < 0.001.

**Table 2:** Initial soil fertility status of trial site.

Soil Property	Value
pH	5.9
Olsen P (mg·kg^−1^)	42
Sulphate S (mg·kg^−1^)	16
Organic S (mg·kg^−1^)	8
CEC (cmol_c_·kg^−1^)	15
Exchangeable Ca (cmol_c_·kg^−1^)	7.5
Exchangeable Mg (cmol_c_·kg^−1^)	0.57
Exchangeable K (cmol_c_·kg^−1^)	0.23
Exchangeable Na (cmol_c_·kg^−1^)	0.18
Base Saturation (%)	60.6
Total C (% *w*/*w*)	2.85
Total N (% *w*/*w*)	0.25
C/N Ratio	11
Anaerobic Mineralizable N (kg·N·ha^−1^) ^1^	160

^1^ Measured by anaerobic incubation [23].

## Abbreviations

The following abbreviations are used in this manuscript:

DCD	Dicyandiamide
DM	Dry matter
N	Nitrogen
